# Impact of hydration with beverages containing free sugars or xylitol on metabolic and acute kidney injury markers after physical exercise

**DOI:** 10.3389/fphys.2022.841056

**Published:** 2022-10-20

**Authors:** Wojciech Wołyniec, Andrzej Szwarc, Katarzyna Kasprowicz, Katarzyna Zorena, Marta Jaskulak, Marcin Renke, Marta Naczyk, Wojciech Ratkowski

**Affiliations:** ^1^ Division of Occupational, Metabolic and Internal Diseases, Medical University of Gdańsk, Gdańsk, Poland; ^2^ Department of Sport Sciences, Gdańsk University of Physical Education and Sport, Gdańsk, Poland; ^3^ Faculty of Physical Education, Gdańsk University of Physical Education and Sport, Gdańsk, Poland; ^4^ Division of Immunobiology and Environmental Microbiology, Medical University of Gdańsk, Gdańsk, Poland; ^5^ Laboratory of Nutritional Biochemistry, Department of Clinical Nutrition, Medical University of Gdańsk, Gdańsk, Poland; ^6^ Department of Athletics, Gdańsk University of Physical Education and Sport, Gdańsk, Poland

**Keywords:** sweetener, polyols, soccer, physical exercise, hypohydration, hyperuricemia, acute kidney injury

## Abstract

The proper fluid and carbohydrates intake is essential before and during physical exercise, and for this reason most athletes drink beverages containing a high amount of free sugars. Sweetened soft drinks are also commonly consumed by those not doing any sport, and this habit seems to be both unhealthy and also the cause of metabolic problems. Recently, several sweeteners have been proposed to replace sugars in popular beverages. To examine the impact of free sugars and the popular sweetener xylitol on metabolic profile and the markers of kidney function and injury after exercise the present study was conducted with semi-professional football players. All participants were healthy, with a mean age of 21.91 years. Their sports skills were on the level of the 4th-5th division of the league. The subjects took part in four football training sessions. During each session they drank a 7% solution of sugar (sucrose, fructose, glucose) or xylitol. The tolerability of these beverages and well-being during exercise was monitored. Before and after each training session, blood and urine were collected. The markers of kidney function and injury, uric acid, electrolytes, complete blood count, CRP, serum albumin, serum glucose and the lipid profile were analyzed. The main finding of this study was that the xylitol beverage is the least tolerated during exercise and 38.89% of participants experienced diarrhea after training and xylitol intake. Xylitol also led to unfavorable metabolic changes and a large increase in uric acid and creatinine levels. A mean increase of 1.8 mg/dl in the uric acid level was observed after xylitol intake. Increases in acute kidney injury markers were observed after all experiments, but changes in urine albumin and cystatin C were highest after xylitol. The other three beverages (containing “free sugars” - glucose, fructose and sucrose) had a similar impact on the variables studied, although the glucose solution seems to have some advantages over other beverages. The conclusion is that sweeteners are not a good alternative to sugars, especially during exercise. Pure water without sweeteners should be drunk by those who need to limit their calorie consumption.

**Clinical Trial Registration:** ClinicalTrials.gov, (NCT04310514)

## 1 Introduction

It is commonly believed that physical activity is beneficial and constitutes the best preventive measure in numerous diseases (Pedersen and Saltin, 2015). However, doing sport, especially at the professional level, is associated with certain health risks (Scheer et al., 2021). The major ones pertain to the musculoskeletal and cardiovascular systems. The adverse impact of physical exercise on the kidneys is less known and less explored (Hoffman and Weiss, 2016). Renal damage following physical exertion is relatively rare, and is most often the consequence of severe hypohydration, muscle damage or rapid weight loss (Sawka et al., 2007; Garcia-Arroyo et al., 2020; Juett et al., 2020; Krisher et al., 2020; Juett et al., 2021).

Hypohydration is one of the most important complications after physical activity, especially when it is strenuous and performed in high temperatures (Armstrong et al., 2007; Seo et al., 2014). In individuals performing high-intensity exercise in heat, the sweating rate can be as high as 1–2.5 liters per hour. In this situation, sweat output can easily exceed voluntary fluid/water intake, which rarely reaches 1 liter per hour during exercise (Sawka and Montain, 2000; Sawka et al., 2007). Therefore, a body water deficit and electrolyte losses are commonly observed after physical activity (Armstrong et al., 2007 (Sawka and Montain, 2000).

Hypohydration greater than 2% of body mass impairs performance during endurance exercise, decreases physical work capacity and has a negative impact on mood (Sawka and Montain, 2000; Sawka et al., 2007; Seo et al., 2014; Bibiloni et al., 2018; García-Berger et al., 2020). The risk of rhabdomyolysis, exercise-associated hyponatremia and acute kidney injury (AKI) is increased in subjects with a body water deficit (Krisher et al., 2020; Juett et al., 2021).

Generally, during exercise, drinking in accordance with thirst is recommended, especially in moderate climates, when sweat output is relatively low (Sawka & Montain, 2000; Sawka et al., 2007). It is reasonable advice for amateur athletes, but drinking according to thirst could be inefficient in professionals. Therefore, precise recommendations have been proposed for professional footballers. To minimize the risk of dehydration, pre-hydration before football matches and adequate fluid intake during breaks is recommended by UEFA experts. Players should ingest 5–7 ml/kg of their body mass of fluid in the 2–4 hours prior to the match (Sawka et al., 2007; Collins et al., 2021). FIFA recommended special cooling breaks—additional breaks for cooling and drinking (Dvorak and Junge, 2015).

In sports, ingestion of carbohydrates helps to maintain a proper glucose level in the blood and to delay the exhaustion of glycogen deposits. Professional football players have great energy demands, because during the match. Energy expenditure is as high as 1,300–1,600 kcal (Seo et al., 2014). It has been accepted by sports experts that it is appropriate to consume a drink comprising carbohydrates (CHO) and electrolytes (Sawka et al., 2007; Marcos et al., 2014; García-Berger et al., 2020; Millard-Stafford et al., 2021). Players should consume 1–3 g of CHO/kg body mass 3–4 h before kick-off and it is recommended to consume 30–60 g CHO after the warm-up and again at half-time (Collins et al., 2021). The preferable sugars are glucose, sucrose and fructose, which are easily ingestible and are called free sugars (Ibrahim, 2018; Swan et al., 2018). In modern societies, the consumption of soft drinks is increasing rapidly, especially in children and teenage populations. A high intake of beverages containing sugar leads to metabolic diseases such as obesity, diabetes and hyperuricemia, cardiological, and nephrological complications, hypertension and cancers. (Johnson et al., 2010; Singh et al., 2015; Bratoeva et al., 2017; Johnson et al., 2018; Garcia-Arroyo et al., 2020; Zafar, et al., 2021). In order to reduce the incidence of cardio-metabolic diseases, the WHO suggests controlling body weight by limiting calorie intake and a frequent strategy for this is to reduce the consumption of drinks containing free sugars and replacing sugars in the diet with low or non-caloric substitutes called alternative sweeteners (Popkin and Hawkes, 2016). Plenty of “good” or “healthy” sugars as well as “harmless” sweeteners are added to beverages. They improve the taste of these beverages and have a very low glycemic index. They are also recommended by some authorities, albeit with some caution (Argiana et al., 2020; Ashwell et al., 2020). Polyols are hydrogenated carbohydrates used as sugar substitutes. One of the most popular and commonly used is xylitol (Livesey, 2003). Xylitol is not used by professional sportsmen. They need energy from CHO and do not look for sugar substitutes. Otherwise, xylitol is an alternative sweetener for overweight and obese amateur sportsmen, who look for ‘healthy’ nutrition.

In this study, we compare the impact of free sugars and xylitol on metabolic profile and kidney function. To the best of our knowledge, no paper concerning this problem has been published yet.

## 2 Materials and methods

### 2.1 Participants

All participants were males, healthy amateur football players who agreed to participate in the study. Their sports skills were on the level of 4-5th division of the league. All declared no chronic disease and none were taking medicines for chronic and acute medical problems during the study (Table 1).

**TABLE 1:** Baseline characteristic of subjects who completed all experiments.

Participants (*n* = 11)	Results (mean ± SD)
Age (years)	21.91 ± 1.30
Height (cm)	183.82 ± 4.49
Weight (kg)	86.81 ± 11.23
BMI (kg/cm^2^)	25.65 ± 2.68

Abbreviation. BMI, body mass index.

The group of football players had been chosen due to methodological reasons. All participants had very similar age, anthropometric features, training experience and were able to perform the same exercise every week.

The inclusion criteria were: readiness to participate, signed written informed consent, age between 20 and 30 years. The exclusion criteria were: a history of kidney disease, serum creatinine >1.2 mg/dl, eGFR <60 ml/min/1.73 m2 and an albumin-to-creatinine ratio (ACR) > 15 mg/g or hematuria in urinalysis before the study.

Due to the complicated format of the study, which was performed between waves of the COVID-19 pandemic, only a proportion of the participants completed all experiments. Thirty-one participants, all male (mean age: 22.5 years; 20–25), were included in the first part of the study. Eventually, 24 participants completed at least 2 training sessions. In this group, the tolerance of various beverages and self-reported well-being were analyzed. Thirteen participants completed all 4 experiments. Two subjects from this group had a creatinine level above 1.2 mg/dl before the study and their biochemical results were not analyzed.

### 2.2 Study design

The study was a randomized, double-blind, crossover study with each subject serving as their own control by completing all four beverage experiments**.**

Before the first experiment, one investigator (WW) drew lots, taking a paper tickets from a container. Each participant received their own number (from 1 to 31). Participants received subsequent beverages according to a previously prepared schedule.

All experiments were conducted in the morning, 1–2 h after breakfast. The participants were asked to record their dietary intake, including all beverages. They were allowed to eat a light breakfast according to their own habits. However, they were asked not to drink sodas, soft drinks or other sweetened beverages. These meals typically consisted of bread, ham, cheese or milk with cereals. Participants were also asked to replicate a similar dietary intake on subsequent days. This approach—the same meal according to the participant’s own habits - was chosen to minimize the effect of a breakfast on the biochemical results. Training without a meal would be poorly tolerated. The calculations of meal contents are shown in Table 2, as well as the percentage of dietary recommendations for the general population and football players.

**TABLE 2:** The analysis of subjects breakfasts.

Component	Quantity mean ± SD
Calories (kcal)	262.98 ± 189.30
CHO (g)	34.59 ± 20.98
CHO/kg BW (g/kg)	0.43 ± 0.28
CHO according to UEFA recommendations^a^ (%)	43.11 ± 28.38
CHO according to DC/AND/ACSM recommendations^b^ (%)	16.70 ± 10.49
Protein (g)	11.81 ± 10.12
Fat (g)	8.08 ± 9.52
Fiber (g)	2.48 ± 1.85
Fluid intake (ml)	182.14 ± 122.78
Fluid/kg BW (ml/kg)	2.46 ± 1.57
Fluid according to UEFA recommendations*** (%)	51.54 ± 30.15

a At least 1 g CHO/kg BW, before exercise.

b At least 200 g CHO, 2–4 h before exercise; ***at least 5 ml/kg BW, of fluid 2–4 h before exercise.

Abbreviations. CHO, carbohydrates; BW, body weight; UEFA, Union of European Football Associations; DC, Dietitians of Canada; AND, Academy of Nutrition and Dietetics; ACSM, the American College of Sports Medicine.

### 2.3 Trainings

The study subjects participated in football training sessions resembling a typical match (two 40-min training units with a 15-min half-time break). These sessions took place every week in the pre-season training period. The participants were asked to refrain from strenuous activities during the 24 h period prior to each test.

In the four subsequent training sessions, during the half-time break, the participants each time received a different drink, although this was always a 7% solution of sugar (containing 35 g of sucrose or 35 g of glucose or 35 g of fructose) or sugar substitute (35 g of xylitol). The order of drink administration was not known to the participants and it was different for each player. 5 g of citric acid was added to all beverages to improve the taste, which was the same for all fluids. The water used to prepare beverages was low mineralized water containing calcium (108 mg/L), sodium (2.5 mg/L), magnesium (1.6 mg/L), potassium (1.1 mg/L), bicarbonates 267 (mg/L), sulfur 25 (mg/L), fluorides (<0.1 mg/L) and chlorides (6.3 mg/L).

The beverages were prepared before the experiments by one researcher (WW). All drinks were in plastic bottles and appeared to be identical. Each bottle was marked with the subject's given name and, in some cases, the first letter of family name. There was no information about the content of beverages on the bottles. The list of subsequent beverages was not attached to them. Only one researcher (WW) had a list. The drinks were delivered by the trainers (WR, AS), who did not know what kind of beverage was in the bottle. The order in which participants received drinks was in line with a previously prepared schedule. Beverage acceptability and gastrointestinal comfort were monitored and reported in the participant questionnaires.

### 2.4 Blood and urine samples

Blood and urine samples were collected before and after all trainings. Blood was drawn from the antecubital vein in a sitting position by experienced nurses. The most of the measurements were performed immediately after experiments in certified laboratory. The blood was centrifuged at 1500 G for 10 min (AFI-C600RE*-*2P*-*AUTO KATRINA). Urine samples were centrifuged 1000 G for 5 min (Sigma Laboratory Centrifuges 3K18). Part of the urine sample was frozen up and stored at −80°C for up to 6 months before analysis of biomarkers of acute kidney injury new marker.

### 2.5 Measurements

The part of measurements (biochemical, blood count, urinalysis, and urine albumin) were performed in local commercial laboratory. The laboratory workers did not know what drinks were used during experiments. The part of urine was frozen and after 6 months the measurements of new makers of AKI were performed by two investigators (MJ & KZ) from Medical University of Gdansk. MJ and KZ had no information which beverage was used during subsequent trainings.

#### 2.5.1 Biochemical measurements

Serum and urinary creatinine and urea were measured by a Kinetic Assay (Alinity c Creatinine, Urea Reagent Kit, Abbott Laboratories, Abbot Park, IL, United States). Serum and urinary uric acid was measured by Enzymatic Assay on Abbott automated clinical chemistry analyzers (Alinity c Uric Acid Reagent Kit, Abbott Laboratories). Serum and urinary sodium, potassium and chloride were measured using ion-selective electrodes for Na, K, and Cl by using Cobas 8,000 analyzer (Roche Diagnostics GmbH). Serum and urinary calcium was measured by Calcium Arsenazo III Colorimetric Method (Alinity c Calcium Reagent Kit, Abbott Laboratories). Serum and urinary phosphate were measured spectrophotometrically by molybdate assay (Alinity Phosphorus Reagent Kit, Abbott Laboratories).

Serum albumin was measured by a colorimetric method (Alinity c Albumin BCG Reagent Kit, Abbott Laboratories). C-reactive protein (CRP) was measured in serum using a Cobas 8,000 analyzer (Roche Diagnostic GmbH, Mannheim). Serum glucose was measured by enzymatic method (Alinity c Glucose Kit, Abbott Laboratories). Total cholesterol was measured by enzymatic method (Alinity c Cholesterol Reagent Kit, Abbott Laboratories). Triglycerides were measured by using enzymatic reagent glycerol phosphate oxidase (Alinity c Triglyceride Reagent Kit, Abbott Laboratories). The high-density lipoprotein cholesterol (HDL) was measured by accelerator selective detergent (Alinity c Ultra HDL Reagent Kit, Abbott Laboratories). The low-density lipoprotein cholesterol (LDL) was measured by liquid selective detergent (Direct LDL Regent Kit, Abbott Laboratories).

#### 2.5.2 Hemoglobin and blood cells count

Hemoglobin was measured by spectrophotometry with the sodium lauryl sulphate (SLS)-hemoglobin method using Sysmex XN 2000 (Sysmex Europe GmbH, Norderstedt, Germany). Platelets were measured by the direct current (DC) detection method with Sysmex XN 2000 (Sysmex Europe GmbH). Leucocytes were measured by flow cytometry Sysmex XN 2000 (Sysmex Europe GmbH). All reagents used in the Sysmex XN 2000 analyzer came from Sysmex Europe GmbH.

#### 2.5.3 Urinalysis and urine albumin

Before and after experiment the urine was analyzed by using a ten-patch test strip for the semi-quantitative determination of specific gravity, pH, leukocytes, nitrite, protein, glucose, ketone bodies, urobilinogen, bilirubin, and blood (Cobas 411 analyzer, Roche Diagnostics GmbH, Mannheim, Germany). Elements contained in urine were measured using the flow cytometry method (UF 1000i, SYSMEX EUROPE GmbH, Norderstedt, Germany). Urinary albumin was measured by an Immunoturbidimetric Assay (Alinity c Microalbumin Reagent Kit, Abbott Laboratories).

#### 2.5.4 Determination of urine human lipocalin-2/NGAL, TIM-1/KIM-1/HAVCR, and cystatin C concentrations

Urine concentrations of human lipocalin-2/NGAL, TIM-1/KIM-1/HAVCR, and Cystatin C were measured using the immunoenzymatic ELISA method (Quantikine High Sensitivity Human by R&D Systems, Minneapolis, Minn, United States) according to the manufacturer’s protocol. Minimum and maximum detectable concentrations were determined by the manufacturer as 0.012–40 ng/ml, 0.009–10 ng/ml, and 0.102–200 ng/ml, respectively.

Intraassay (3.6% for lipocalin-2/NGAL; 4.3% for TIM-1/KIM-1/HAVCR; 6.6% for Cystatin C) and interassay (7.9% for lipocalin-2/NGAL; 6.3% for TIM-1/KIM-1/HAVCR; 7.0% for Cystatin C) respectively. The absorbances were read on the automated plate reader ChroMate 4,300 (Awareness Technology, Inc, United States) at the wavelength λ = 450 nm. The reference curve was prepared according to the manufacturer’s recommendations.

### 2.6 Reference values

The complete list of reference values can be found in the Supplementary File S1.

### 2.7 Calculations and equations

The complete list of calculations and equations can be found in the Supplementary File S2.

### 2.8 Location and time

All training sessions took place in the temperate climate in the north of Poland, 24 m above sea level. Four training sessions were performed on 10, 17, 24 and 31 May 2021 in the morning hours (8.30–10.30).

### 2.9 Weather condition

The weather conditions during each session were similar, and typical for spring in the temperate climate (Table 3).

**TABLE 3:** Weather conditions.

Date	Temperature	Dew point	Humidity	Wind speed (km/h)	Atmospheric pressure (hPa)	Precipitation (mm)	Condition
10.05.2021	14°C–19°C	6°C	59%–43%	19–24	995.54–994.56	0.0	Fair
17.05.2021	14°C–17°C	9°C–11°C	72%–68%	4–15	986.69	0.0	Fair
24.05.2021	12°C–16°C	8°C–6°C	77%–52%	11–19	1001.44–1002.43	0.0	Fair—Partly Cloudy
31.05.2021	12°C–14°C	2°C–3°C	51%–48%	15–19—15	1007.35	0.0	Fair

### 2.9 Statistical analyses

The authors used Statistica 12 software (StatSoft, Kraków, Poland) for the analysis. The Shapiro-Wilk test was applied to assess homogeneity of dispersion from the normal distribution. When the Shapiro-Wilk’s test showed a normal distribution, the paired t-test was used. Where the Shapiro-Wilk’s test showed that the distributions of the examined parameters were significantly different from normal (*p* < 0.05), the non-parametric Wilcoxon Signed Rank test was used. Descriptive statistics for continuous variables were reported as mean values and standard deviations. Cochran’s Q test was used to compare the distributions of the two qualitative variables.

Two-way analysis of variance (ANOVA) with repeated measures (group [fructose vs. glucose vs. sucrose vs. xylitol] × Time [pre vs. post]) was used to evaluate beverage effects on selected acute kidney injury and function markers. In case of a significant interaction, Tukey’s post hoc test was performed to assess differences in particular subgroups. In all analyses, a *p*-value lower than 0.05 was considered statistically significant.

## 3 Results

### 3.1 Hydration before

The hydration status was analyzed in 24 subjects who completed at least two experiments. Before training sessions 18% of participants were well hydrated, 27% mildly hypohydrated and 55% hypohydrated, according to urine specific gravity measurements.

### 3.2 Beverage tolerance and well-being

The questionnaires concerning gastrointestinal problems and well-being were analyzed in 24 subjects who completed at least two training sessions. 50% (12/24) of subjects had at least one gastrointestinal problem after at least one training session. Diarrhea was the most common problem and was reported by almost 39% of subjects. This was the only statistically significant difference between the groups. The type of beverage had no statistical impact on participants’ well-being. 26% of subjects reported better well-being after sucrose, and almost 16% worse well-being after xylitol, but these changes did not reach a level of statistical significance (Table 4).

**TABLE 4:** Beverage tolerance and well-being.

	All (%)	Fructose (%)	Glucose (%)	Sucrose (%)	Xylitol (%)	*p*
Abdominal pain	7.4	0	0	11.1	22.2	ns
Nausea	8.6	0	0	22.2	16.7	<0.05
Diarrhea	16	5.6	0	27.8	38.9	<0.01
Makes me feel good	—	18.2	16.7	26.1	10.5	ns
Makes me feel bad	—	0	1.5	13	15.8	ns

Abbreviations: ns, non significant.

### 3.3 Changes in urea, uric acid and creatinine serum levels and urine excretion

After all training sessions, creatinine and uric acid (UA) levels increased significantly in serum, but serum urea was unchanged. The highest increases in creatinine and UA were observed after xylitol intake. After 7% xylitol beverage, a mean UA increase of almost 2 mg/dl was observed and the mean value of UA exceeded the upper normal limit of UA (the normal range is up to 7.2 mg/dl). The increase in creatinine was significant, but the post-exercise values did not exceed normal values (Supplementary File S1). The rate of creatinine and UA level increases was very similar after xylitol intake. Therefore the UA-to-creatinine ratio was calculated. This ratio was unchanged after xylitol, but decreased significantly after 7% glucose beverage intake. This means that after glucose intake the increase in UA was lower than the increase in creatinine. Both the fractional excretion in urea (FeUrea) and uric acid (FeUA) decreased significantly after all experiments. Both these values reflect decreased renal blood flow (RBF). There was no increase in urea, which was untypical in exercise, and in this experiment was probably related to good hydration during exercise and the relatively low intensity of the exercise. All results are presented in a Table 5.

**TABLE 5:** Changes in urea, uric acid and creatinine serum levels and urine excretion.

	Fructose			Glucose			Sucrose			Xylitol		
	pre	post	*p* value	pre	post	*p* value	pre	post	*p* value	pre	post	*p* value
sCr (mg/dl)	0.92 ± 0.06	1.06 ± 1.18	<0.001	0.92 ± 0.06	1.05 ± 0.07	<0.00001	0.95 ± 0.07	1.11 ± 0.07	<0.001	0.93 ± 0.07	1.24 ± 0.14	<0.0001
sUrea (mg/dl)	31.55 ± 5.47	31.36 ± 5.5	ns	30.91 ± 8.48	31 ± 8.43	ns	31.2 ± 6.78	31.6 ± 6.74	ns	29.64 ± 4.63	30.82 ± 4.69	ns
FeUrea (%)	36.07 ± 7.00	28.17 ± 6.48	<0.01	38.66 ± 7.84	31.61 ± 11.04	<0.01	38.77 ± 7.80	27.18 ± 8.13	<0.001	35.9 ± 5.86	26.27 ± 5.9	<0.00001
sUA (mg/dl)	5.83 ± 0.99	6.51 ± 0.97	<0.0001	5.76 ± 1.04	6.2 ± 0.98	<0.001	6.03 ± 0.90	6.83 ± 0.95	<0.001	5.93 ± 1.07	7.88 ± 1.34	<0.0001
FeUA (%)	5.12 ± 1.53	3.51 ± 0.73	<0.01	4.81 ± 1.16	3.65 ± 0.92	<0.05	5.16 ± 1.23	3.43 ± 0.89	<0.01	4.53 ± 1.05	2.72 ± 0.65	<0.0001
sUA/Cr ratio (mg/mg)	6.32 ± 1.11	6.2 ± 1.1	ns	6.28 ± 1.22	5.92 ± 0.93	0.007	6.37 ± 0.95	6.12 ± 0.64	ns	6.38 ± 1.10	6.41 ± 1.10	ns

Abbreviations: sCr-serum creatinine; sUrea, serum urea; sUA, serum uric acid; FeUrea, fractional urea excretion; FeUA, fractional uric acid excretion; sUA/Cr, serum uric acid to creatinine ratio; ns, non significant.

Two-way ANOVA revealed that the increase in uric acid and creatinine was also related to the type of beverage. The increase in creatinine was higher after the xylitol beverage than after the glucose and fructose ones. The increase in serum UA was higher after xylitol compared to glucose. All results are presented in a Table 6.

**TABLE 6:** The influence of different interventions on serum biomarkers (two-way ANOVA).

Variable	Effect	F	p	Post hoc outcome
sCr (mg/dl)	Group	7.25	<0.001	X. > F; X.> G
	Time (pre vs. post)	115.12	<0.000001	post > pre
	Group*time	6.28	<0.001	F2 > F1, G1, S1 X1; G2 > F1, G1, X1
				S2 > F1, G1, S1, X1; X2> F1, G1, S1 X1
				X2 > F2, G2, S2
sUA (mg/dl)	Group	3.284	<0.05	X > G
	Time (pre vs. post)	18.527	<0.0001	post > pre
	Group*time	2.329	ns	
FeUrea (%)	Group	1.111	ns	
	Time (pre vs. post)	30.276	<0.000001	pre > post
	Group*time	0.367	ns	
FeUA (%)	Group	2.135	ns	
	Time (pre vs. post)	48.721	<0.000001	pre > post
	Group*time	0.409	ns	

Abbreviations: sCr-serum creatinine; sUA, serum uric acid; FeUrea, fractional urea excretion; FeUA, fractional uric acid excretion. F, fructose; G; glucose, S -sucrose; X, xylitol; 1, before training, 2, after training; ns, non significant.

### 3.4 Estimation of renal blood flow and hypohydration

A mild increase in sodium and a decrease in the potassium level and fractional excretion (FeK) were not significant after all experiments. Statistically significant decreases in FeNa and uNa/K and an increase in uK(K + Na) were found. All these changes are typical for reduced RBF. USG did not change significantly. The mild increase in albumin was significant in all but one group. There was no change in albumin during hydration with 7% glucose. The increase in albumin is typical for hypohydration and hemoconcentration. The lack of any change after glucose intake suggests good hydration after this sugar. Two-way ANOVA revealed that increase of serum albumin and uK(K + Na) ratio and decrease uNa/K ratio were related to time but not type of beverage (Table 7).

**TABLE 7:** Changes in markers of renal blood flow and dehydration.

	Fructose			Glucose			Sucrose			Xylitol		
	pre	post	*p* value	pre	post	*p* value	pre	post	*p* value	pre	post	*p* value
Na (mM/L)	140.82 ± 2.56	140.82 ± 2.96	ns	139.36 ± 3.14	139 ± 3.03	ns	140.9 ± 0.99	142.1 ± 1.37	ns	141.64 ± 1.80	142.18 ± 1.78	ns
K (mM/L)	4.47 ± 0.38	4.23 ± 0.31	ns	4.23 ± 0.28	4.19 ± 0.43	ns	4.55 ± 0.39	4.34 ± 0.41	ns	4.33 ± 0.32	4.29 ± 0.42	ns
FeNa (%)	0.44 ± 0.22	0.24 ± 0.16	<0.01	0.57 ± 0.52	0.38 ± 0.5	<0.05	0.44 ± 0.26	0.22 ± 0.24	<0.01	0.33 ± 0.23	0.18 ± 0.15	<0.01
FeK (%)	7.31 ± 4.46	7.17 ± 2.98	ns	6.34 ± 3.16	6.19 ± 2.02	ns	5.34 ± 2.74	5.89 ± 2.02	ns	6.38 ± 3.17	5.99 ± 2.88	ns
uNa/K (mM/mM)	2.10 ± 1.11	1.08 ± 0.63	<0.01	3.32 ± 3.04	1.75 ± 1.59	<0.05	3.23 ± 2.96	1.11 ± 0.8	<0.05	2.02 ± 1.44	1.02 ± 0.48	<0.01
uK(K + Na) (mM/mM)	0.36 ± 0.11	0.515 ± 0.13	<0.001	0.31 ± 0.14	0.44 ± 0.17	<0.0001	0.32 ± 0.15	0.53 ± 0.16	<0.00001	0.39 ± 0.15	0.53 ± 0.16	<0.001
USG	1.0250 ± 0.00465	1.02627 ± 0.00369	ns	1.02146 ± 0.00647	1.02245 ± 0.00695	ns	1.02536 ± 0.0042	1.0279 ± 0.00284	ns	1.02618 ± 0.00379	1.02736 ± 0.00301	ns
Serum albumin (mg/mL)	46.64 ± 2.11	47.64 ± 1.86	<0.05	46.45 ± 1.44	47.27 ± 1.49	ns	46.3 ± 2	47.7 ± 1.7	<0.05	46.82 ± 1.4	49.27 ± 1.10	<0.0001

Abbreviations: USG, urine special gravity, ns-non significant.

### 3.5 AKI biomarkers

Urinary albumin and cystatin C increased in all groups, reaching the highest values after xylitol intake. Both cystatin C and albumin are reabsorbed in the proximal tubule, and these changes probably reflect reduced proximal tubule reabsorption. When albumin and cystatin C were normalized to creatinine, significant changes in ACR were found in all groups, but uCysC/Cr was increased only after xylitol intake. Urinary NGAL increased only after sucrose and xylitol, but when normalized to creatinine, it was unchanged in all groups. Urinary KIM increased after sucrose and fructose but when normalized to creatinine, it decreased. The decrease in uKIM/Cr can be explained by enhanced creatinine excretion during exercise and reflects the difficulty in interpreting AKI markers after exercise (Table 8).

**TABLE 8:** Changes in urinary acute kidney injury biomarkers.

	Fructose			Glucose			Sucrose			Xylitol		
	pre	post	*p* value	pre	post	*p* value	pre	post	*p* value	pre	post	*p* value
uAlb (mg/L)	14.197 ± 9.924	80.36 ± 150.47	<0.01	12.50 ± 6.17	38.67 ± 34.11	<0.05	12.86 ± 6.61	86.17 ± 113.98	<0.01	15.77 ± 9.24	161.92 ± 287.01	<0.01
ACR (mg/g)	5.5 ± 1.69	20.7 ± 36.94	<0.001	6.23 ± 2.40	12.5 ± 9.42	<0.001	5.01 ± 1.6	20.79 ± 28.88	<0.0001	5.35 ± 2.20	37.48 ± 67.12	<0.001
uNGAL (ng/mL)	18.79 ± 11.52	22.4 ± 10.87	ns	21.71 ± 13.19	22.95 ± 10.42	ns	12.99 ± 12.41	21.2 ± 12.73	<0.05	9.33 ± 8.28	21.99 ± 12.06	<0.01
uNGAL/uCr (μg/g)	8.32 ± 5.07	6.74 ± 3.68	ns	12.96 ± 11.24	12.58 ± 16.33	ns	5.96 ± 6.10	5.24 ± 3.01	ns	4.32 ± 5.98	5.73 ± 3.58	ns
uKIM-1 (ng/ml)	1.18 ± 0.64	1.61 ± 1.28	<0.05	1.21 ± 0.88	1.34 ± 1.14	ns	0.95 ± 0.51	1.35 ± 1.02	<0.05	1.37 ± 1.02	2.11 ± 1.96	ns
,uKIM/uCr (μg/g)	0.55 ± 0.34	0.46 ± 0.29	<0.05	0.55 ± 0.27	0.39 ± 0.2	<0.01	0.39 ± 0.19	0.32 ± 0.2	ns	0.48 ± 0.23	0.48 ± 0.33	ns
uCyst-C (ng/ml)	51.91 ± 21.73	92.37 ± 42.8	<0.01	42.25 ± 22.6	75.62 ± 40.7	<0.01	46.25 ± 26.33	80.89 ± 41.37	<0.01	46.84 ± 21.65	103.34 ± 43.01	<0.001
uCyst/uCr (μg/g)	22.75 ± 7.95	26.42 ± 7.51	ns	22.16 ± 12.22	26.3 ± 9.12	ns	18.99 ± 7.59	20.61 ± 7.34	Ns	18.58 ± 7.97	27.06 ± 10.05	<0.05

Abbreviations: uAlb, urinary albumin; ACR, albumin-to-creatinine ratio; uNGAL, urinary neutrophil gelatinase-associated lipocalin; uKIM-1–kidney injury molecule 1; uCyst-C–urinary cystatin C., uNGAL/uCr-urinary NGAL to creatinine ratio, uKIM/uCr urinary KIM-1 to creatinine ratio, uCyst/uCr -urinary cystatine C to creatinine ratio. ns-non significant.

### 3.6 Calcium and phosphate serum levels and urine excretions

A serum calcium increase and a urinary calcium excretion decrease were noticed after all experiments. There was a positive correlation between serum concentration and urinary excretion of calcium. Serum phosphate decreased after all experiments, except the one with a glucose beverage. Excretion of phosphate decreased after all experiments, except the one with a xylitol beverage. All results are shown in a Table 9.

**TABLE 9:** Changes in calcium and phosphate serum levels and urine excretions.

	Fructose			Glucose			Sucrose			Xylitol		
	pre	post	*p* value	pre	post	*p* value	pre	post	*p* value	pre	post	*p* value
Calcium (mg/dl)	9.67 ± 0.37	9.95 ± 0.27	<0.01	9.66 ± 0.22	9.95 ± 0.25	<0.01	9.55 ± 0.30	9.84 ± 0.33	<0.05	9.56 ± 0.19	10.15 ± 0.24	<0.0001
Phoshpate (mg/dl)	3.74 ± 0.657	3.327 ± 0.674	<0.05	3.51 ± 0.52	3.12 ± 0.47	ns	3.79 ± 0.61	3.18 ± 0.48	<0.001	3.70 ± 0.66	3.15 ± 0.59	<0.01
uCa/Cr (mg/mg)	0.06 ± 0.05	0.03 ± 0.02	<0.01	0.09 ± 0.11	0.05 ± 0.04	<0.01	0.06 ± 0.05	0.03 ± 0.02	<0.01	0.05 ± 0.02	0.03 ± 0.02	<0.01
FePi (%)	11.26 ± 5.46	6.4 ± 3.50	<0.01	10.64 ± 4.28	5.9 ± 3.36	<0.001	12.7 ± 2.94	8.6 ± 4.39	<0.01	9.36 ± 4.24	8.58 ± 5.74	ns

Abbreviations: uCa/Cr, urine calcium to creatinine ratio; FePi, fractional excretion of phosphate; ns, non significant.

### 3.7 Glucose and lipids

The changes in lipids were observed mainly after xylitol beverage intake. The statistically significant change in the glucose level was found only after 7% glucose beverage (Table 10).

**TABLE 10:** Changes in glucose and lipids.

	Fructose			Glucose			Sucrose			Xylitol		
	pre	post	*p* value	pre	post	*p* value	pre	post	*p* value	pre	post	*p* value
Glucose (mg/dl)	93.46 ± 9.26	99.82 ± 11.74	ns	81.55 ± 8.24	95.18 ± 13.31	<0.01	85.36 ± 11.83	98.18 ± 14.92	ns	89.27 ± 15.32	101.18 ± 10.52	ns
Cholesterol (mg/dl)	149.27 ± 16.76	146.64 ± 18.41	<0.05	152.8 ± 20.18	150.5 ± 21.53	ns	154.9 ± 23.85	153.00 ± 25.53	ns	147.6 ± 19.79	149.8 ± 21.1	<0.05
TG (mg/dl)	109.27 ± 32.58	91.55 ± 34.19	ns	101.2 ± 30.83	102.7 ± 31.1	ns	92.4 ± 35.09	84.9 ± 27.04	ns	88.7 ± 36.79	77.8 ± 27.37	ns
LDL (mg/dl)	77.27 ± 18.52	77.91 ± 17.43	ns	78.82 ± 23.91	76.73 ± 22.46	ns	86.1 ± 25.41	84.1 ± 26.3	ns	77.5 ± 18.58	81.9 ± 18.84	<0.05
HDL (mg/dl)	50.09 ± 9.80	50.46 ± 9.00	ns	49.00 ± 10.17	48.46 ± 9.49	ns	50.20 ± 11.21	51.8 ± 11.03	<0.05	52.4 ± 10.68	53.7 ± 10.03	<0.05

Abbreviations: TG, triglycerides; LDL, low-density lipoprotein; HDL, high-density lipoprotein; ns, non significant.

### 3.8 Hematocrit, blood cells count and CRP

There was no change in the CRP level, and only mild changes in the hematocrit and blood cell count were found. All results are shown in a Table 11.

**TABLE 11:** Changes in hematocrit, blood cells count and CRP.

	Fructose			Glucose			Sucrose			Xylitol		
	pre	post	*p* value	pre	post	*p* value	pre	post	*p* value	pre	post	*p* value
Hct (%)	46.473 ± 2.71	45.11 ± 2.64	<0.01	45.76 ± 2.96	44.62 ± 2.44	ns	46.33 ± 2.67	45.03 ± 2.27	<0.01	46.28 ± 2.43	45.96 ± 2.56	ns
WBC (G/L)	6.205 ± 1.032	6.29 ± 1.29	ns	5.54 ± 0.9	5.38 ± 1.01	ns	6.47 ± 1.37	6.47 ± 1.80	ns	6.2 ± 0.83	6.8 ± 1.99	ns
Hgb (g/dl)	15.60 ± 1.117	15.27 ± 0.956	<0.05	15.34 ± 1.07	15.11 ± 0.86	ns	15.7 ± 1.03	15.42 ± 1.0	ns	15.65 ± 0.80	15,62 ± 0.95	ns
PLT (G/L)	232.18 ± 45.02	241.36 ± 49.64	ns	225.27 ± 38.21	238.55 ± 39.65	<0.01	235.45 ± 47.85	242.73 ± 47.51	ns	229.64 ± 40.8	241.18 ± 47.11	<0.05
CRP (mg/L)	1.88 ± 2.34	1.88 ± 2.28	ns	1.41 ± 0.65	1.36 ± 0.63	ns	1.35 ± 0.48	1.35 ± 0.48	ns	1.5 ± 1.0	1.56 ± 1.1	ns

Abbreviations: Hct, hematocrit; WBC, white blood count; Hgb, hemoglobin; PLT, platelets; CRP, c-reactive protein; ns, non significant.

## 4 Discussion

The main finding of this study was that the 7% xylitol solution is the worst tolerated beverage during exercise and leads to unfavorable metabolic changes and a large increase in uric acid and creatinine levels. The other three beverages (containing “free sugars” - glucose, fructose and sucrose) had a similar impact on the variables studied, although the glucose solution seems to have some advantages over other beverages.

The participants experienced more gastrointestinal problems after consuming sucrose drinks than fructose or glucose, but a much larger proportion said the drink “makes me feel good”. These findings are difficult to explain. We used a 7% solution of sugars which is the highest recommended concentration. For some athletes even a 7% solution can cause some disturbances. Monosaccharides are slightly better absorbed than disaccharide, since the latter must first be hydrolyzed. This could be the reason for better tolerance of a high dose of monosaccharide.

The participants were generally pleased when they received drinks during the break, because they were not used to drinking half a liter during this time. It appears that proper hydration makes them feel good, but a high dose of sugars can cause some discomfort.

### 4.1 Hydration

The proper hydration and carbohydrate intake is essential before exercising and exercise performance is diminished when water and calorie intake are inappropriate (Russell et al., 2012). Various sports drinks are recommended not only to prevent dehydration but also to improve athletic performance (Orru et al., 2018). This study shows that in their breakfast habits semi-professionals rarely follow the UEFA recommendations. The subjects consumed less than half of the recommended calories and water. Although this is surprising, those subjects were not professionals and had no knowledge of UEFA recommendations. Interestingly, similar results were obtained in a study of elite soccer players from the Spanish La Liga (Barcelona FC), in this group fewer than 30% of the subjects were euhydrated prior to training sessions (Rollo et al., 2021). This finding is consistent with previous observations that many athletes are poorly hydrated before training sessions (García-Berger et al., 2020). In a study of recreational basketball players, an at all astonishing 17.5% of subjects reported that they did not drink before exercise (Bibiloni et al., 2018). The present study and previous studies show that the awareness of proper fluid intake is low.

The study participants drank 500 ml of a beverage during the break in the middle of training. This session took place in a moderate climate and was not very intense, therefore the risk of dehydration was minimal. Estimating hydration status after exercise is difficult. Several biochemical variables which were proposed to assess dehydration in subjects at rest have little value during exercise. After exercise, changes in these variables (FeNa, FeK(K + Na), FeUrea, FeUA) mainly reflect a reduction in renal blood flow (RBF), which is a fully physiological process caused by the activation of the renal angiotensin-aldosterone system and increased sympathetic tone (Juett et al., 2020). Hypohydration, if it does occur, can exacerbate this reduction and cause pre-renal acute kidney failure. In the present study, changes in all RBF markers were observed. The highest albumin increase, which is typical for hemoconcentration and dehydration, was observed in the xylitol group, but there were no changes in hematocrit and hemoglobin typical for hypohydration.

### 4.2 Acute kidney injury

The diagnosis of acute kidney injury (AKI) after physical exercise is problematic (Poussel et al., 2020). The increase in creatinine can be explained by muscle metabolism and damage and several biochemical changes routinely observed in clinical setting after physical activity only reflect a physiological decrease in RBF (Wołyniec et al., 2018). In this study, markers of AKI were studied and showed the highest rise in the xylitol group. Markers of proximal tubule dysfunction (cystatin C and albumin) were particularly elevated after exercise and xylitol intake. The consequences of these mild changes are undefined.

### 4.3 Sugar and its substitutes

In many countries, rapidly increasing consumption of soft drinks is reported, followed by alarming information about the effects of sugars. Sugar-sweetened beverages (SSB) are soft drinks, carbonated sodas, juice drinks with added sugar, energy drinks, and sports drinks (Popkin and Hawkes, 2016). The increased intake of high-calorie beverages leads to many health problems, such as obesity, diabetes and metabolic syndrome or post-exercise airway narrowing (Cao et al., 2016; Emerson et al., 2016). The high consumption of sugars, together with a low intake of water, leads to chronic kidney problems (Garcia-Arroyo et al., 2020).

Physical exercise is an important tool in preventing and treating metabolic diseases, although its effects depend on the duration and intensity of physical activity (Donnelly et al., 2009; Lee et al., 2022; Ruegsegger and Booth, 2018).

Many recreational and amateur sportsmen do sports to reduce body mass. During physical activity, they are encouraged to drink the appropriate volume of fluids but many sports drinks have sugar additives. These beverages are tasty, therefore for some subjects it is difficult to stop consuming them and to drink only water. Since the consumption of high amounts of sugars is unhealthy, many sugar substitutes have been proposed as ingredients of “zero” or “low” sugar drinks (Smeets et al., 2011; Qurrat-ul-Ain and Khan, 2015). Among the most commonly used sweeteners are polyols (xylitol, erythritol, sorbitol, mannitol, maltitol, isomalt, lactitol, and polyglycitol). They have a low glycemic index (GI), low insulinemic index and are non-cardiogenic. They are also low-digestible and one of their main adverse effects is osmotic diarrhea after oral administration of these sugars. This adverse effect is, however, for some subjects helpful and expected (Wang and van Eys, 1981; Livesey, 2003; Meyer-Gerspach, 2021). In order to decrease the incidence of metabolic and cardiovascular diseases, the WHO suggests limiting calorie intake. One of the strategies is to replace sugars in the diet with low or non-caloric alternative sweeteners. Sugar substitutes are recommended for people with diabetes due to their low glycemic index and positive impact on metabolic parameters, although this is done with some caution (Argiana et al., 2020; Ashwell et al., 2020).

### 4.4 Xylitol

In the present study, we found a large increase of uric acid level after xylitol intake. Xylitol is one of the most popular polyols and has a 10 times lower glycemic index than glucose. Xylitol is advertised as a natural food and an ingredient of “eco-friendly” recipes. Since xylitol is believed to be safe, no limit in its daily intake has been specified (Gasmi Benahmed et al., 2020; Takayasu et al., 2020). It is an approved food additive used as a sweetener (dietary supplement, toothpaste, chewing gum). The anti–carcinogenic properties of xylitol and its positive effect on oral hygiene are clear and worth emphasizing (Gasmi Benahmed et al., 2020); Wang and van Eys, 1981).

However xylitol is a popular sweetener there is a lack of new studies concerning acute impact of xylitol ingestion on metabolism and kidney function.

### 4.5 Xylirol and gastrointestinal problems

Beverages containing CHO or xylitol with the addition of citric acid have almost the same taste, which was acceptable for all the football players studied. All beverages used in the study were well tolerated, although after exercise some subjects experienced gastrointestinal problems. The most common complaint was diarrhea, reported by almost 40% of subjects after xylitol intake. This is not surprising, because only 50% of xylitol is absorbed from the intestine (Livesey, 2003). During physical activity, this property of xylitol is undesirable. It diminishes the ability to perform sport, and leads to poorer hydration. The additive used in sports drinks (sugars, salts) should increase water absorption whereas xylitol works inversely. The analysis of biochemical variables also suggests that hydration status after xylitol was the worst.

### 4.6 Xylitol and uric acid

A rapid increase in uric acid levels was observed after intake of xylitol during exercise. Generally, an increase in uric acid after exercise is related to enhanced metabolism and diminished renal excretion. It is known that foods with a higher content of sugars, fructose, fat and purine bases, starvation and dehydration lead to a UA increase (Hamada et al., 2008). In recently published studies after ingestion of a nucleotide-rich meal the UA level increased by 12% and after 7 days of a high-nucleotide diet by 60% (Coelho, et al., 2020; Coelho, et al., 2022).

In our study we found that after exercise and xylitol intake the UA level increased by 33% and the mean value of UA exceeded its upper limit. It is rather uncommon to observe an increase of 2 mg/dl in serum UA in such a short period of time.

Previously published papers suggest that an increase in UA after xylitol is related to increased purine degradation, although data concerning the relationship between xylitol and purines are rather scarce. In 1977, Forster found that xylitol used as a sugar substitute for 4 weeks in children with diabetes led to an increase of 1 mg/dl in serum uric acid concentration (Förster et al., 1977). The same author studied 12 healthy volunteers consuming xylitol (40–100 g/day for 18 days) or sucrose (6 days, 80–100 g/day) and reported only few cases of diarrhea at the beginning of the study as an adverse event (Förster et al., 1982). All the studies cited above were published many years ago and awareness of the possible impact of xylitol on uric acid metabolism is still rather unknown.

It is not clear to what degree the increase in UA presented in this study was related to increased purine degradation and to what extent to a decrease in renal blood flow. It is also not clear what impact is made by a short-lasting acute increase of uric acid. An acute increase in uric acid leads to acute kidney injury (e.g. after chemotherapy) (Hahn et al., 2017), but the impact on health of an acute UA increase after exercise is unknown.

## 5 Conclusion

During the 2014 World Cup, soccer fans could observe a new approach to the problem of proper hydration. Special cooling breaks were introduced to allow players to drink during hot days. Additionally, during Euro 2020 Championships, the players’ choices of water and commercial beverages were widely commented (Reuters Agency, 2021). The media concentrated on the financial consequences of some players’ statements, but the physiological considerations are more interesting. The question what to drink is not simple and differs between sports, weather conditions and professional level. For professional players, a relatively large volume of fluids and a high amount of carbohydrates are recommended, because during intensive exercise, it is difficult to stay properly hydrated and maintain the high intensity of exercise.

Amateur and recreational sportsmen do not have to follow all recommendations for professionals. For amateurs, water, the first and best beverage for humankind (Wolf et al., 2008) should be recommended. The worst approach is to use sweeteners instead of sugars. They give a false signal to the brain, and lead to unfavorable metabolic changes.

## Data Availability

The raw data supporting the conclusions of this article will be made available by the authors, without undue reservation. And I did not detect any particular expressions.
