# Supplementary material for: Impact of hydration with beverages containing free sugars or xylitol on metabolic and acute kidney injury markers after physical exercise
Source: Front Physiol. 2022 Oct 20;13:841056. doi: 10.3389/fphys.2022.841056 (PMC9632281; doi:10.3389/fphys.2022.841056)
Supplement: Supplementary file 1 [file DataSheet2.PDF]

Supplementary file 2.

1.1 Calculations and Equations

1. Hydration status was define as:

subjects euhydrated: urine specific gravity (USG) < 1.020,

minimally hypohydrated: USG 1.021-1.024;

hypohydrated USG > 1.024

2. Fractional excretions of uric acid (FeUA), urea (FeUrea), sodium (FeNa), potassium (FeK), phosphate (FePi) were calculated using the following formula:

Fractional excretion of a parameter (%) = (urine parameter x serum creatinine)/(serum parameter x urine creatinine)

3. Serum uric acid to creatinine (sUA/Cr) ratio was calculated using the following formula:

Uric acid to creatinine ratio = serum uric acid ÷ serum creatinine.

4. Urinary sodium to potassium ratio was calculated using the following formula:

uNa/K ratio = urinary sodium ÷ urinary potassium.

5. Urinary potassium to sodium + potassium ratio was calculated using the following formula:

uK / (K+Na) ratio = urinary potassium ÷ (urinary potassium + urinary sodium).

6. Urinary calcium to creatinine ratio was calculated using the following formula:

uCa/Cr = urinary calcium ÷ urinary creatinine.

7. Albumin to creatinine ratio (ACR) in urine was calculated using the following formula:

ACR (mg/g) = urine albumin/urine creatinine.
