# Supplementary material for: Impact of hydration with beverages containing free sugars or xylitol on metabolic and acute kidney injury markers after physical exercise
Source: Front Physiol. 2022 Oct 20;13:841056. doi: 10.3389/fphys.2022.841056 (PMC9632281; doi:10.3389/fphys.2022.841056)
Supplement: Supplementary file 2 [file DataSheet1.PDF]

Supplementary file 1.

Table. 1.1. Normal values of biochemical variables and blood count

Variable	Unit	Reference ranges (male)
sCr	mg/dL	0.72 - 1.25
sUrea	mg/dL	19 - 44
sUA	mg/dL	3.5 - 7.2
Na	mmol/L	136 - 145
K	mmol/L	3.5 - 5.1
Calcium	mg/dL	8.4 - 9.7
Phoshpate	mg/dL	2.3 - 4.7
Glucose	mg/dL	70 - 99
Cholesterol	mg/dL	115 - 190
TG	mg/dL	< 150
LDL	mg/dL	< 115
HDL	mg/dL	> 40
CRP	mg/L	< 5.0
Serum albumin	g/L	35 - 52
uAlb	mg/L	< 20
ACR	mg/g	< 30
Hct	%	40 - 52
WBC	G/L	4.0 - 11.0
Hgb	g/dL	13.0 - 18.0
PLT	G/L	150 - 400

Abbreviations: sCr- serum creatinine, sUrea – serum urea, sUA – serum uric acid, TG – triglycerides, LDL- low-density lipoprotein, HDL – high-density lipoprotein, CRP – c-reactive protein, uAlb - urinary albumin, ACR–albumin-to-creatinine ratio, Hct – hematocrit, WBC – white blood count, Hgb – hemoglobin, PLT – platelets,

Table. 1.2. Reference values of novel urinary biomarkers for renal damage

**Variable**	**Unit**	**Reference value (male; 21-30 years old)** (Pennemans, et al., 2013)
uNGAL	ng/mL	< 73.88
uNGAL/uCr	μg/g	< 125.5
uKIM-1	ng/mL	< 1.86
uKIM/uCr	μg/g	< 2.28
uCyst-C	ng/mL	< 208.2
uCyst/uCr	μg/g	< 220

Abbreviations: uNGAL–urinary neutrophil gelatinase-associated lipocalin, uKIM-1 – kidney injury molecule 1, uCyst-C–urinary cystatin C., uNGAL/uCr-urinary NGAL to creatinine ratio, uKIM/uCr urinary KIM-1 to creatinine ratio, uCyst/uCr - urinary cystatine C to creatinine ratio.
